# Vitamin B_12_ deficiency as a cause of severe neurological symptoms in breast fed infant – a case report

**DOI:** 10.1186/s13052-020-0804-x

**Published:** 2020-03-30

**Authors:** Cezary Dubaj, Katarzyna Czyż, Wanda Furmaga-Jabłońska

**Affiliations:** Department of Neonate and Infant Pathology, Medical University of Lublin, Prof. Gębali 6 str, 20-093 Lublin, Poland

**Keywords:** Breast-feeding, Infant, Vitamin B_12_ deficiency, Psychomotor retardation, Megaloblastic anemia, Addison-Biermer disease

## Abstract

**Background:**

Vitamin B_12_ (cobalamin, cbl) deficiency in children is rare and may occurs in exclusively breast fed infants of mothers on vegetarian or vegan diet with lack of appropriate supplementation. The clinical manifestation of vitamin B12 deficiency include neurological disorders, megaloblastic anemia and failure to thrive. Routine and commonly used laboratory tests such as cell blood count (CBC) or serum vitamin B_12_ level are sufficient for appropriate diagnosis. Typical therapy is based on intramuscular cobalamin injections. Early diagnosis and early onset of treatment are crucial factors for long-term prognosis of patients as the duration of deficiency may be correlated with the development of long lasting changes in the nervous system.

The purpose of this article is to present influence of maternal vitamin B_12_ deficiency as a cause of infant psychomotor retardation.

**Case presentation:**

We report the case of a 7 months old girl whose parents sought medical advice due to pathological somnolence and developmental regression of their daughter with onset approximately 2 months prior to the visit. Following several diagnostic tests it was determined that the infant’s symptoms were due to vitamin B_12_ deficiency which was secondary to the mother’s latent Addison-Biermer disease. Apart from neurological symptoms the infant also showed megaloblastic anemia which is typical to cobalamin deficiencies. Intramuscular vitamin B_12_ supplementation resulted in instant improvement of the patient’s general condition and blood morphology. Unfortunately, psychological examination indicated long-term psychomotor retardation due to delayed diagnosis of B_12_ deficiency.

**Conclusions:**

Vitamin B_12_ levels should be considered during differential diagnosis of neurological symptoms in exclusively breast-fed infants especially if they co-exist with megaloblastic anemia and psychomotor retardation.

## Background

Vitamin B_12_ (cobalamin, cbl) deficiency in children is usually manifested by a number of non-specific symptoms such as developmental retardation, irritability, reduced muscle tone or difficult weaning, which definitely obstructs diagnosis. Megaloblastic anemia coexisting with neurological symptoms may indicate a deficiency related cause with primary focus given to folic acid deficiency [1, 2]. When such symptoms are observed it is important to extend the diagnosis and verify vitamin B_12_ levels as its deficiency may affect especially those infants who are fed exclusively with breast milk [2, 3]. Vitamin B_12_ therapy results in instant improvement while long-term deficiency may cause permanent damage to the nervous system [4]. It is usually related to the mother’s megaloblastic anemia, vegetarian or vegan diet with lack of appropriate supplementation [5, 6]. Since the popularity of such diets is increasing and a significant proportion of mothers feed their infants exclusively with breast milk for the first 6 months, a further increase in incidence of the cobalamin deficiency may be expected. It is also significant that the symptoms are manifested much sooner in the infant than in the mother, which may cause difficulties in the diagnostic process and may mislead the physicians to look for other causes. Routine and commonly used laboratory tests such as cell blood count (CBC) or serum vitamin B_12_ level are sufficient for appropriate diagnosis [4, 5]. The presented case is a 7 months old female infant with severe neurological symptoms, diminished consciousness and activity, somnolence and long-term regression of psychomotor development which were caused by cobalamin deficiency.

## Case presentation

The girl aged 6 months and 26 days feeding exclusively with breast milk was admitted to the Department of Neonate and Infant Pathology Medical University in Lublin due to symptoms of psychomotor development regression and increased somnolence with onset approximately 2 months earlier. The infant was born of second pregnancy complicated with maternal hyperthyroidism, with normal birth parameters (gestation age 40 weeks, birth weight 3010 g, length 50 cm, Apgar score: 10). Neurological examination at admission indicated severe somnolence and abnormal muscle tone which was diminished in trunk, neck and lower extremities. In the upper extremities muscle tone was increased with fine muscle tremor. Deep reflexes were quite vivid and symmetrical. Verbal and visual contact with the infant was possible but significantly limited. According to the mother, the infant was active only for about 30 min a day, slept for approximately 20 h a day and the activity during the remaining 3–4 h was significantly reduced. The mother reported that the child was developing normally and appropriately for her age group until the age of 5 months. She used to be very active, able to change position from prone to supine and vice versa. In the prone position she used to extend the lower extremities, raised her head and forearms and was able to stabilise her head when pulled up to a sitting position. While sitting, she was able to maintain balance with minimum external support, reached towards objects but was unable to hold an object in each hand simultaneously. She responded to smile, started to babble, produced a number of sounds including shouts of joy and turned her head towards a person who called her name.

At the admission to the hospital certain diagnostic procedures were done to reveal reasons of infant’s condition. Laboratory test results indicated megaloblastic anemia red blood cells count (RBC) – 2.95 × 10^6^/μL norms:3,7–6.00 × 10^6^/μL, hematocrit - 26.8%, norms: 33.0–40.0%, hemoglobin level 9.2 g/dL norms:10.5–13.5 g/dL, mean corpuscular volume (MCV) of red blood cell - 90 fl norms: 74–89 fl, mean cell haemoglobin (MCH) 31.2 pg. norms:24–30 pg. Thyroid hormone level was within normal limits (TSH – 1.785 mU/L, FT3–6.01 pmol/L, FT4–16.35 pmol/L) and other biochemical blood test results did not identify significant abnormalities. Toxoplasma gondi and Cytomegaly virus infections antibody level were excluded, as well. Neurological results and CBC prompted further tests for folic acid level (result 16.32 ng/mL, reference range above 5.38 ng/mL) and B_12_ serum level (result 75 pg/mL; reference range 211–911 pg/mL). Due to the low cobalamin blood level in infant, diagnosis was extended to rule out Addison – Biermer disease and gastric anti-parietal cell antibodies level was assessed (APCA) – the result of the test was negative. Screening for metabolic diseases by tandem MS was also negative. Organic acid urine level tested showed increased methylmalonic acid concentration (4460 mg/g of creatinine; reference range up to 50 mg/g. Performed EEG was within normal limits, cranial MRI showed slightly dilated cerebro-spinal fluid spaces in the temporal and frontal regions.

Following hospital admission, psychological examination using the Brunet – Lezine Scale (psychomotor development in early childhood) was conducted in order to evaluate the infant’s developmental status in the following areas: P – posture and locomotion, C – visual-motor coordination, L – language and S – social and emotional interactions plus overall assessment [7–9]. The scale allows to determine both the child’s developmental age (DA) separately in each of the areas, to define the developmental profile and to calculate partial and total developmental quotient (DQ). DQs level between 85 and 115 indicate an average developmental level and are defined as normal limits. A score of 84–70 is considered borderline and indicates developmental status below average. Quotient below 70 means retardation of definite psychomotor area or total development.

The total psychomotor development level of the patient at admission to hospital and prior to vitamin B_12_ therapy was significantly retarded as compared to her actual age (her calendar age was 6 months and 26 days but the evaluated developmental age was 3 months and 20 days; DQ = 54). Visual-motor coordination (C) development was evaluated at 2 months and 20 days, the infant was not able to pursuit moving objects with her eyes, the range of eye movements was 90 degrees (reference value is 180), she did not try to reach towards objects did not seem interested in the environment (DQ = 39). Posture and locomotion development (P) was retarded (suitable for 4 months and 10 days), the girl was unable to sit on her own, required strong support when seated and her muscle tone was reduced (DQ = 63). Social and emotional interactions (S) were at the level of a 4 months old as the infant did not react with facial expressions to interactions (IR = 58). Speach and language development (L) was rated as low as 2 months and 10 days since the infant produced only few consonants (DQ = 34, see Table 1).

**Table 1:** Evaluation of psychomotor development using Brunet-Lezine scale before and after vitamin B_12_ supplementation [7–9]

Psychomotor development areas	Before vitamin B_**12**_ supplementation Calendar age: 6 months 26 days		After vitamin B_**12**_ supplementation Calendar age: 16 months	
	Developmental age (DA) months;days	Developmental quotient (DQ)	Developmental age (DA) months;days	Developmental quotient (DQ)
Posture and locomotion (P)	4;10	63	12;0	75
Visual-motor coordination (C)	2;20	39	13;24	86
Social and emotional reactions (S)	4;0	58	12;0	75
Language skills (L)	2;10	34	12;0	75
**Total development**	3;20	54	12;09	77

Developmental Age (DA) of the child is a total of the scores from each area P, C, L, S, which is then divided by 10.

DA = P + C + L + S/10

Total Developmental Quotient (DQ) is calculated as the developmental age (DA) divided by the child’s calendar age (CA) both values expressed in days, and then multiplied by 100.

Total DQ = (DA/CA) × 100

Since the infant was fed exclusively with breast milk, the mother B_12_ serum level was verified and the result was slightly below the lower limit e.g.189 pg/mL. The mother was not on any exclusive diet and consumed animal products. Diagnostic tests in mother did not detect anemia, CBC result was normal (RBC 4.49 × 10^6^/μL, hematocrit 43.1%, hemoglobin 14.5 g/dL), but mean cellular volume (MCV) and mean cell haemoglobin (MCH) was elevated, that is: 95.9 fl and 32.3 pg respectively. In the mother’s medical history there was no information of any gastro-intestinal disease. The mother underwent extended diagnostic tests with detected APCA, which were positive and led to the diagnosis of latent Addison-Biermer disease. The mother receives vitamin B_12_ supplementation (200 μg intramusculary once a month through the rest of her life) and normal B_12_ blood levels are maintained.

Based on the anamnesis and laboratory results e.g. low vitamin B_12_ level and significantly increased methylmalonic acid urine level, the infant was diagnosed with cobalamin deficiency. Substitution treatment was start immediately: vitamin B_12_ intramuscular injections 50 μg twice a week, in total 5 doses during 2,5 weeks were administered The infant’s condition improved significantly during cobalamin supplementation. She became much more active, stayed awake longer, started reaching out for toys and smiling, and her eye contact has improved. Milk formulas were introduced and the patient’s diet was extended as per the age. Treatment evaluation conducted 6 weeks later showed further progression of psychomotor development, also somnolence and abnormal muscle tone in the extremities were eliminated. Blood count confirmed normal RBC parameters. Control vitamin B_12_ level was 501 pg/mL.

Psychological evaluation at the age of 16 months (calendar age) i.e. after 10 months following vitamin B_12_ treatment showed psychomotor retardation with results slightly below normal (approx. at the level of 13 months; DQ = 77). Posture and locomotion (P): the child was able to stand unsupported, started to take first steps but still required being held by both hands, bent down to pick up toys while standing, crawled upstairs, climbed low furniture (DQ = 75, equivalent to the age of 12 months). Visual-motor coordination development (C) was equivalent to the age of 13 months 24 days (lower normal limit), she developed pincer grip, was able to hold an object in each hand and reached for another one. She was able to manipulate objects, checked their properties but was unable to fit shapes (circle, triangle, square) into a puzzle box, spontaneously doodled without demonstration (DQ = 86). Language skills (L): vocabulary consisted of 3 words, which was at the level of a 12 months old infant (DQ = 75). Social and emotional interactions (S) were developed at the level of a 12 month old, the infant was very cheerful and trustful, responded to smile, sought interactions, understood bans, handed over objects when asked and shown, repeated activities when she was smiled at (DQ = 75, see Table 1).

During 10 months after treatment vitamin B_12_ level was 622 pg/mL and remained stable throughout the follow up period.

## Discussion and conclusions

The first description of vitamin B_12_ (cobalamin cbl) deficiency in infants was published in 1962 by Jadhav et al. [10] based on six cases of infants aged 7–12 months with developmental regression and megaloblastic anemia. Subsequent publications described similar symptoms as in our patient caused by cobalamin deficiency which were quickly relieved by substitution treatment. All cases described in literature, including the case discussed below, were fed exclusively with breast milk. This is due to the fact that the most common reason of cobalamin deficiency in infant is secondary to maternal deficiency caused by the vegetarian diet or disrupted absorption of cobalamin from the gastrointestinal tract [11, 12]. This is usually related to maternal Addison-Biermer disease as in our study [4, 11, 12]. Cobalamin absorption requires binding to an intrinsic factor (IF, also referred to as the Castle factor) produced by gastric parietal cells and the vitamin B_12_-IF complex is absorbed in the final section of the small intestine. Although Addison-Biermer disease is referred to malignant anemia, the disease does not necessarily coexist with anemia, as in the presented paper.

The daily demand for vitamin B_12_ in neonates and infants is between 0.1 and 0.4 μg. This demand is perfectly satisfied by the average cobalamin concentration in breast milk (approx. 0.42 μg/L), provided that there is no maternal deficiency [11]. Therefore even short-term changes in maternal B_12_ metabolism subsequently lead to deficiency of cobalamin in infant due to low B_12_ concentration in breast milk [12].

Symptoms of cobalamin deficiency in breast fed infants are usually manifested 4 to 8 months after birth [6]. Delayed diagnosis may be related to the lack of specific symptoms, especially in subclinical forms. Appropriate diagnosis should be based on megaloblastic anemia coexisting with neurological symptoms such as developmental regression, apathy, hypotonia or reduced contact with the child. It should be noted however, that anemia may be normocytic with cooccurrence of iron deficiency. Oftentimes it is also associated with pale yellowish coloration of skin and atrophy of lingual papillae. Another significant symptom is difficult weaning and vomiting following intake of foods other than breast milk [1, 6]. In case of co-occurrence of the above symptoms and megaloblastic anemia, the vitamin B_12_ serum level should be verified. Certain researchers suggest that diagnostics should be extended with tests for methylmalonic acid level in urine or blood [13]. The concentration of methylmalonic acid is a very sensitive indicator of cobalamin metabolism, especially in case of subclinical disorders, when the serum level of cobalamin or red blood parameters are within normal ranges. Cobalamin acts as a cofactor in the conversion from methylmalonyl-CoA to succinate-CoA so in case of cobalamin deficiency, methylmalonic acid is accumulated in the body what was presented in our study [4, 11, 14].

Typical therapy for vitamin B_12_ deficiency in infants is based on intramuscular cobalamin injections. Various plans may be found in literature with different treatment regimens and dosage schemes [15]. Additional supplementation using iron preparations may be considered in order to prevent the development of iron deficiency, as the demand for iron increases significantly due to intensified hematopoiesis [6]. Clinical case study shows that immediate improvement of the infant’s condition is achieved within 5–7 days following the onset of vitamin B_12_ therapy. In our patient the improvement is most prominent in reduced apathy and somnolence as well as normalised muscle tone and appetite [4–6]. Biochemical and hematology results tests have also improved significantly (mainly RBC level and MCV).

Vitamin B_12_ is known to take part in two reactions in the human body. The first is homocysteine methylation to methionine in which vitamin B_12_ acts as a co-factor. In the second reaction cobalamin participates in the conversion of methylmalonyl-CoA to succinate-CoA. Vitamin B_12_ deficiency slows down this reaction and causes accumulation of precursor compounds including methylmalonyl-CoA in organism, which was found in the presented case. According to Frenkel [16], vitamin B_12_ deficiency leads to synthesis of abnormal fatty acids which become part of neuronal myelin sheaths structure causing neurological disorders. The most common theories explaining neuropathy secondary to cobalamin deficiency were focused on the reactions mentioned above with methylmalonic acid and homocysteine being perceived as neurotoxins acting on the central and peripheral nervous systems [4, 5]. However, the available study results did not show any correlation between the concentration of the above substances and the severity of changes in the nervous system [17].

Neurodegenerative symptoms related to vitamin B_12_ deficiency are described in the available publications as subacute encephalopathy which mainly affects the white matter. Histologically vitamin B_12_-dependent encephalopathy may be characterised as spongiform vascuolisation, intramyelinic and interstitial edema of the white matter, of the axons and brain with axonal damage especially in spinal cord [18]. In rats subject after full gastrectomy, the mentioned changes in the central nervous system caused a significant reduction in nerve signalling with profound reduction of white matter density [17, 18].

Some researchers claim that cobalamin does not directly influence the pathophysiology of the nervous system but it may affect the balance of cytokines responsible for myelin sheath metabolism [19, 20]. According to Scalabrino et al. [19] certain cytokines such as tumor necrosis factor-α (TNF-α) or nerve growth factor (NGF) show neurotoxic properties, however others, such as epidermal growth factor (EGF) or interleukin 6 (Il-6) show neuroprotective effects. Experimental studies have shown that cytokine activity is directly dependent on B_12_ metabolism. Cobalamine deficiency cause increased activity of TNF-α with simultaneous reduction of EGF activity, which results in damage to myelin sheaths [20].

Research using mice models conducted by Scalabrino and Veber [21] showed that the damage to myelin sheaths caused by cobalamin deficiency are also associated with increased levels of another protein in the central nervous system, so called normal prions. Elevated level of these proteins may cause neurodegenerative symptoms. Normal prions are physiological membrane glycoproteins which are commonly found in the cells of living organisms [21, 22]. Their main know property is that the conformation of their isoforms may have infectious character and lead to neurodegenerative changes [21–23]. According to the literature normal prions play several crucial roles in the human body [22]. They are responsible for creation and maintaining of synapses, transmembrane signalling, participate in the uptake and binding of copper ions, cell adhesion to extracellular matrix, provide protection against oxidative stress and have cytoprotective effect by inhibiting the initiation of programmed apoptosis [22]. However, it has been shown that excessive expression of normal prions may lead to degeneration and vacuolisation of cells within the CNS [23].

The available literature contains only scarce data concerning the duration and extent of histological and functional change regression as a result of vitamin B_12_ therapy [4, 5, 24]. In the discussed case, the patient’s neurological condition has improved relatively quickly within 1 week of treatment but the psychological evaluation conducted 9 months after hospitalisation still indicated deficits in various areas of psychomotor development. This observation confirms persistent changes in the central nervous system of the child. In the literature there are limited data on long term development after severe neuropathological symptoms in infantile cobalamin deficiency. Pearson and Turner [2] followed up a child diagnosed at 32 months were found to present intellectual delay at age of 6 years. Similar observation in a 14 months old patient was described by Schenk and al [4]. who observed neurodisability and psychomotor retardation lasting up to second year of life. It should be concluded that infantile B_12_ deficiency may cause long lasting neurodisability even though vitamin B_12_ supplementation leads to rapid resolution of severe neurological symptoms [2, 4].

Case studies described in the literature clearly indicate that vitamin B_12_ levels should be considered during differential diagnosis of neurological symptoms in infants, especially if they co-exist with megaloblastic anemia and psychomotor retardation [1, 3–6, 24]. The most crucial factors for long-term prognosis are early diagnosis and early onset of treatment, as the duration of deficiency may be correlated with the development of long lasting changes in the nervous system [4, 24]. Unfortunately, the lack of specific symptoms, especially in the subclinical form of the deficiency prior to the development of megaloblastic anemia, presents another challenge in medical diagnosis. Hopefully, this article will raise awareness among the family doctors and paediatricians concerning the need to verify vitamin B_12_ levels in breast fed infants with neurological symptoms and regression of psychomotor development and megaloblastic anemia.

In the paper there was concluded that vitamin B_12_ deficiency in mother induced by latent Addison-Biermer disease without symptoms of megaloblastic anemia may cause long lasting psychomotor retardation in breast-fed infant.

## Data Availability

All clinical data and supporting materials concerning the manuscript are available in case of Editorial request.

## Abbreviations

Cbl: Cobalamin
CBC: Cell blood count
DA: Developmental age
DQ: Developmental quotient
RBC: Red blood cells
MCV: Mean corpuscular volume
MCH: Mean cell haemoglobin
TSH: Thyroid stimulating hormone
FT3: Free triiodothyronine
FT4: Free triiodothyronine
IgM: Immunoglobulin class M
IgG: Immunoglobulin class G
APCA: Anti-parietall cell antibodies
EEG: Electroencephalogram
IF: Intrinsic factor
AGPC: Anti gastric parietal cel
CNS: Central nervous system
